# Factors influencing public participation behavior relating to government microblogs on COVID-19 updates

**DOI:** 10.3389/fpubh.2024.1337107

**Published:** 2024-03-08

**Authors:** Peng Shao, Menglei Li

**Affiliations:** ^1^School of Management, Xi’an Polytechnic University, Xi’an, China; ^2^School of International Economics, Shaanxi Institute of International Trade and Commerce, Xi’an, China

**Keywords:** megacity, government microblog, public participation, COVID-19, confirmed cases

## Abstract

**Introduction:**

During the global COVID-19 pandemic, densely populated megacities engaged in active international exchanges have faced the most severe impacts from both the disease and the associated infodemic. This study examines the factors influencing public participation behavior on government microblogs in these megacities during the pandemic. It guides megacities in disseminating epidemic information, promoting knowledge on epidemic prevention, managing public opinion, and addressing related matters.

**Methods:**

Utilizing the elaboration likelihood model’s central and peripheral routes, drawing on an empirical analysis of 6,677 epidemic-related microblogs from seven Chinese megacities, this study analyses the influence mechanisms influencing public participation behavior and reveals the regulatory role of confirmed case numbers. Meanwhile,a qualitative comparative analysis examines and discusses diferent confgurations of ixn fuential factors.

**Results:**

The study reveals that microblog content richness demonstrates a U-shaped impact on public participation behavior. Conversely, content interaction, content length, and the number of fans positively impact participation, while update frequency has a negative impact. Additionally, the number of new confrmed cases positively regulates the impact of microblog content and publisher characteristics on public participation behavior. Public participation behavior also varies based on publishing time and content semantic features. This study further revealed the different confgurations of influential factors by QCA method.

**Conclusion:**

This study reveals the impact mechanism of the microblog content and publisher characteristics on public participation behavior. It also demonstrates the regulatory role of newly confrmed cases in the way content and publishers’ characteristics influence public participation behavior. This study is of great significance for the operation of government microblogs, the release of emergency information, and the promotion of public participation.

## 1 Introduction

Information technology offers individuals the opportunity to acquire fast and factual information at any time, thereby helping to limit deterioration of a crisis or disaster (1). The development of the COVID-19 pandemic is accompanied by social media information dissemination, which strongly affects people’s behavior and impacts the effectiveness of government countermeasures (2). In the presence of an infodemic, media organizations must convey accurate information about epidemic prevention and disseminate the true epidemic situation to the public (3). Government social media must provide an avenue to communicate crisis information, share the latest news, and counteract rumors concerning public health. Social media is a prominent source of health information, widely accessed and considered one of the most popular platforms for obtaining such information (4). In China, social media platforms, such as Weibo, have become crucial for individuals requiring assistance during the COVID-19 pandemic (5).

Megacities are the regions with the most active population flows and are the most at risk. High-density aviation networks and numerous international personnel exchanges have facilitated COVID-19’s rapid spread worldwide, and many megacities with large populations and active international exchanges have been hardest hit by the epidemic.

Therefore, this study investigates the following:

Q1: What are the characteristics of epidemic-related microblogs released by the governments of Chinese megacities?

Q2: How does the epidemic development trend affect public participation behavior on microblogs?

Q3: How can public participation behavior relating to the government’s microblogs on the epidemic be improved?

Information disclosure is crucial in China’s official address to the COVID-19 pandemic (6). Based on the official Weibo account content of China’s megacities and the confirmed epidemic data released by the National Health Commission of China, this study explores how public response to epidemic information varies under the dynamic zeroing policy. This study’s contributions to the literature are as follows. First, based on the elaboration likelihood model (ELM), this study reveals the impact mechanism of the microblog content and publisher characteristics on public participation behavior. It also demonstrates the regulatory role of newly confirmed cases in the way content and publishers’ characteristics influence public participation behavior. Third, it reveals the impact of the microblog’s release time and semantic characteristics on public participation behavior. Fourth, using a qualitative comparative analysis (QCA) method, different configurations of the influential factors are discussed.

## 2 Conceptual model and research hypotheses

### 2.1 Conceptual model

Public participation refers to the way the public participates in decision-making or realizes demands through interaction and communication with government organizations (7). Generally, the number of likes, comments, and retweets of blog posts are taken as indicators of public participation (8). Chen et al. (9) collected short videos released by healthy Chinese people during the pandemic and found that video length, title, dialogic loop, and content type significantly influenced the level of citizen engagement. In recent years, the dialogic communication theory has been applied to studies on citizen engagement on social media. Dialogic communication refers to the negotiated interchange of ideas and opinions (10). Governments are responsible for risk management and the country’s resilience in health crises (11). In particular, unexpected diseases that spread quickly, like COVID-19, require more information control and timely interaction between governments and the public (12). The government disseminate up-to-date and reliable news through their social media account. This way, the public can access and utilize information to prevent the further spread of infectious diseases, adopt self-protective measures, and make informed decisions (13). Based on the existing literature, this study takes the government Weibo accounts of Chinese megacities as the research object and sets three dimensions to measure public participation behavior on these accounts, namely, the number of likes, comments, and forwards.

The ELM is well constructed and clearly articulates the persuasion process (14). Moreover, the model’s descriptiveness accommodates various outcomes and, hence, can be used to support many situations. The ELM holds that information processing can act via a central or peripheral route (15). In the information systems field, ELM has been used to examine user behavior in the context of the online community (16). Therefore, this study examines the factors affecting public participation behavior on the government social media sites of megacities from the central and peripheral routes. The central route refers to the content characteristics of the Weibo account, including content richness, content interactivity, and content length; the peripheral route refers to the main characteristics of the Weibo publisher, including the number of fans and the frequency of microblog updates. For this study, urban epidemic characteristics were also included in the research model to explore the regulatory role of the number of confirmed cases in the impact of content characteristics and publisher characteristics on public participation behavior.

### 2.2 Research hypotheses

Content posted on social media is typically presented in plain text, pictures, or videos, with the media richness varying from low to high (17). Lee and Xu (18) investigated the tweets of candidates in the 2016 US presidential election and their impact on voters’ reactions. Plain text tweets on Trump’s account were found to be the most popular among the tweets, but picture tweets did not affect likes and retweets. Chen et al. (8) studied the official account data of the Health Commission’s Sina Weibo account during the COVID-19 epidemic and found that the content richness of health information was negatively correlated with public participation. Owing to the word limit, Twitter users usually extend what they want to express by including complementary materials such as images or videos (18). Research into 50 Facebook accounts of local governments in Western Europe showed that pictures were most likely to promote citizen engagement (calculated by the number of followers, likes, retweets, and posts), followed by plain text (19). However, disputes remain regarding the relationship between content richness and public participation. Therefore, we propose:

H1: A positive U-shaped relationship exists between the content richness of social media posts and public participation behavior.

The dialogic communication theory, proposed by Kent and Taylor (20), is used as a basis to study organizational communication on websites. A dialog often follows two principles: the realization of mutual satisfaction and the creation of a common meaning (10). Rybalko and Seltzer (21) investigated how Fortune 500 companies use Twitter to stimulate stakeholder engagement. The ‘@ + user name’ method enables government microblog users to talk directly with other users and quickly interact with each other. People believe that the inclusion of special symbols such as ‘#’ and ‘@ ‘in Facebook posts can effectively promote the effect of public participation (22), and ‘@ symbol’ and ‘hyperlink’ positively affect the public’s microblog comment forwarding behavior. Therefore, we propose:

H2: Content interactivity positively affects public participation behavior.

Zhang et al. (23), hypothesizing that the public is more sensitive to longer information, found a positive impact of the length of Weibo posts on the number of comments and forwarding. Xu and Zhang (24) analyzed 13,322 tweets about #MH370, the Malaysia Airlines Flight 370 disaster, and found that the number of words contained in each post significantly influenced the number of shares. Citizens are more concerned about whether government social media information can satisfy their needs and reduce uncertainty during times of crisis. Xie et al. (25) analyzed citizens’ information-sharing behaviors on Sina Weibo in the context of Chinese public crises and found that citizens were most interested in receiving information about the government’s handling of the crisis and its developments. Existing evidence supports the assumption that long sentences can obtain more shares (24). Therefore, we propose:

H3: The content length positively affects public participation behavior.

Previous studies have confirmed the importance of the number of the authors’ followers in the information diffusion process (26). The information released by government agency accounts is usually professional and authoritative, and these accounts have built a high degree of trust among the public. The number of followers on Twitter is associated with perceived source credibility (27), leading to higher content acceptance (28). When a government agency account has a large number of followers, the information it publishes will be easier to view, forward, and comment on. Therefore, the number of government social media followers can affect public participation on government social media sites to a certain extent. Thus, we propose:

H4: The number of followers positively affects public participation behavior.

As more content is created, the quality of online content loses uniformity, which creates some difficulties for users searching for, and accepting, information (29). Continuous updating of social media content can improve the timeliness of disseminating epidemic-related information. However, in reality, excessive information input can easily lead to public mood disorder and the public’s failure to receive useful information on time. Excessive information can make it almost impossible to process all of it through only the human brain; this phenomenon is otherwise known as ‘information overload’ (30). Once individuals feel stressed by mass information, their motivation to make sense of new information is reduced, and they recoil from exerting extra effort to verify it (31). Accordingly, we propose:

H5: The update frequency negatively affects public participation behavior.

During crises, citizens turn to leaders, expecting prompt and accurate information (32). Miyabe et al. (33) found that people pay timely attention to rumors and tweets during disasters, often disseminating them upon initial exposure. Wang et al. (15) assert that epidemic information spreads rapidly through social networks, with false information posing a critical threat to COVID-19 management. Government microblogs, by focusing on the epidemic and releasing timely information, play a critical role in enabling the public to stay informed, alleviating social panic, and dispelling public doubts. Therefore, we propose:

H6: The number of confirmed cases plays a positive regulatory role in the influence of content characteristics on public participation behavior.

H7: The number of confirmed cases plays a positive regulatory role in the influence of publisher characteristics on public participation behavior.

## 3 Methods

### 3.1 Data collection

According to the city size classification standard issued by the State Council of the People’s Republic of China, urban areas with a permanent resident population exceeding 10 million are classified as megacities. Based on the seventh national population census data, China has seven megacities. This study focuses on these seven megacities, namely, Shanghai, Beijing, Shenzhen, Chongqing, Guangzhou, Chengdu, and Tianjin (Table 1).

**Table 1:** Basic information of megacities.

City	Total population (10,000 persons)	Urban population (10,000 persons)	Land area (square kilometers)	Location
Shanghai	2,487	1987	6,340	East China
Beijing	2,189	1775	16,418	North China
Shenzhen	1749	1744	1998	South China
Chongqing	3,205	1,634	82,370	Southwest China
Guangzhou	1868	1,488	7,249	South China
Chengdu	2094	1,334	14,335	Southwest China
Tianjin	1,387	1,093	11,934	North China

The original data of the government microblog came from the official microblog account of the megacity government certified by the real name of Sina Weibo and were obtained through a data crawler software. The daily data of new confirmed cases in megacities came from the official website of the National Health Commission of China.

**Figure 1:**
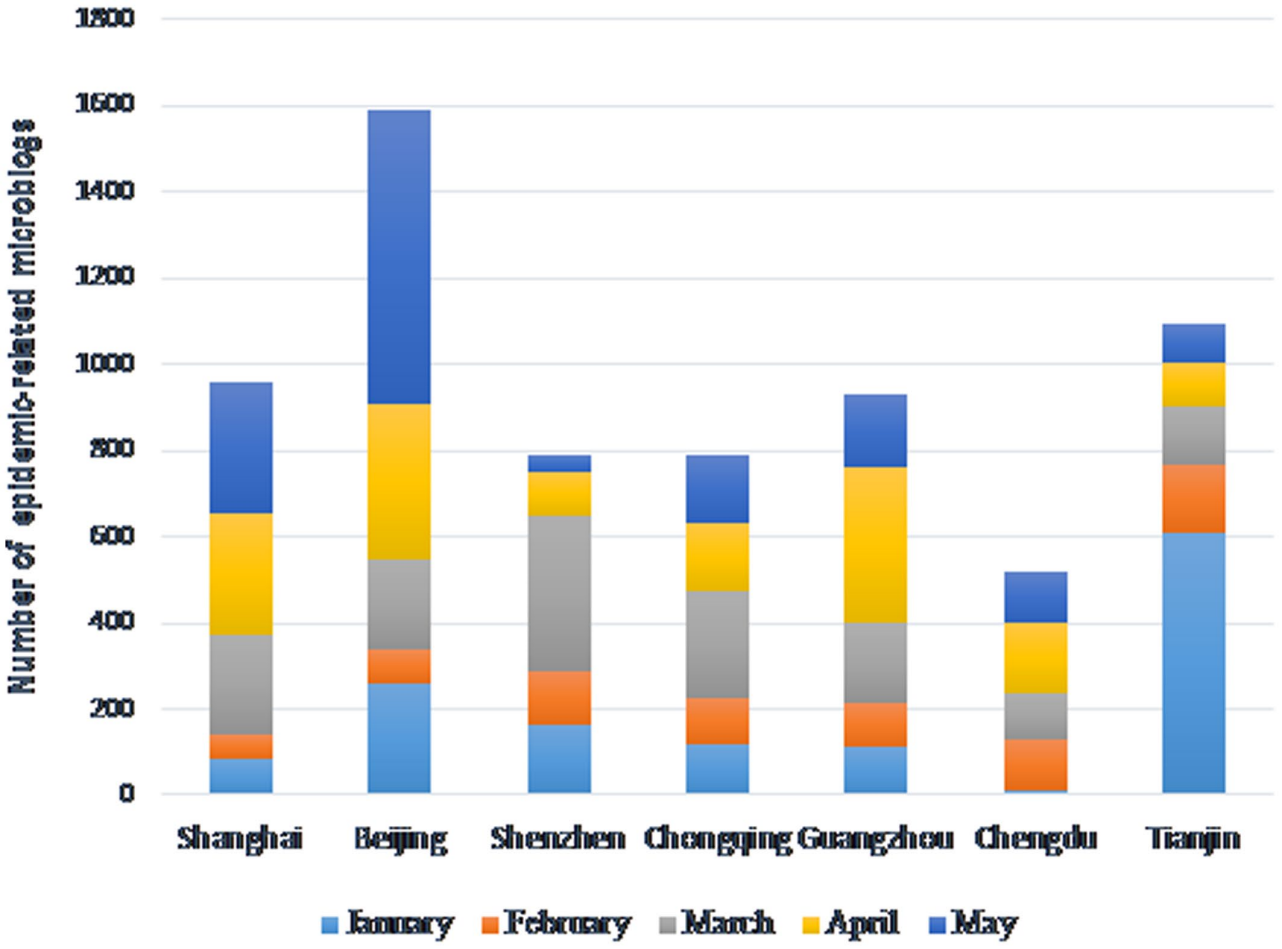
Number of epidemic-related microblogs.

The microblog data covered the period from 1 January 2022 to 31 May 2022 (during which the Chinese government implemented the dynamic zeroing policy), and a total of 24,745 microblogs were obtained. We further cleaned the microblogs using keywords such as ‘epidemic (疫情)’, ‘COVID-19’, ‘pneumonia (肺炎)’, ‘confirmed diagnosis (确诊)’, ‘cases (病例)’, ‘dynamic zeroing (动态清零)’, and ‘vaccines (疫苗)’, and obtained 6,677 microblogs related to the COVID-19 epidemic (see Figure 1).

Among the epidemic-related microblogs, Beijing has the highest count, while Chengdu has the lowest. The peak release month for Beijing was May, Tianjin in January, Guangzhou in April, and Shenzhen in March.

**Figure 2:**
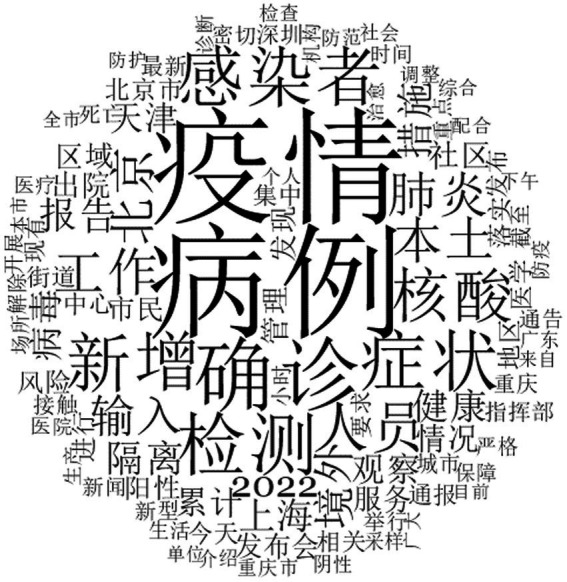
Word cloud of epidemic-related microblogs.

According to the word cloud analysis of the epidemic-related microblog content (see Figure 2), the high-frequency keywords were mainly ‘cases (病例)’, ‘epidemic (疫情)’, ‘confirmed diagnosis (确诊)’, ‘detection (检测)’, ‘symptoms (症状)’, ‘infected individual (感染者)’, and so on. Through high-frequency keyword analysis, we found the collected microblogs suitable for this study (Table 2).

**Table 2:** Word frequency of the 60 most frequent words displayed in Figure 2.

Chinese	English	W.F.	Chinese	English	W.F.	Chinese	English	W.F.
病例	Cases	13,662	上海	Shanghai	3,178	进行	carry out	1815
疫情	Epidemic	13,545	病毒	Virus	3,026	今天	Today	1813
确诊	Confirmed diagnosis	8,452	观察	Observation	2,849	医学	Medicine	1810
检测	Detection	6,927	天津	Tianjin	2,822	相关	Correlation	1784
症状	Symptoms	6,862	累计	Cumulative	2,765	街道	Street	1750
感染者	Infected individual	6,617	情况	Situation	2,540	集中	Focus	1714
新增	Newly	6,568	社区	Community	2,477	发布	Release	1,696
核酸	Nucleic acid	5,830	出院	Discharge	2,347	通报	Notification	1,668
人员	Personnel	5,305	服务	Service	2,276	要求	Requirement	1,581
工作	Work	4,970	管理	Administration	2,263	深圳	Shenzhen	1,570
本土	Mainland	4,678	区域	Region	2,244	最新	Latest	1,564
肺炎	Pneumonia	4,614	阳性	Positive	2,175	现有	Existing	1,560
北京	Beijing	4,530	市民	Citizen	2,128	指挥部	Headquarters	1,545
输入	Input	4,094	发现	Find	2,121	重点	Key point	1,523
境外	Overseas	3,899	发布会	Presentation	2090	死亡	Death	1,522
2022	2022	3,609	风险	Risk	2005	接触	Contact	1,519
隔离	Quarantine	3,385	北京市	Beijing	1874	新闻	News	1,478
报告	Report	3,381	落实	Practice	1825	重庆	Chongqing	1,473
措施	Measure	3,318	中心	Core	1822	截至	As of	1,471
健康	Health	3,195	地区	Region	1816	密切	Close	1,468

W.F., Word Frequency.

### 3.2 Operationalization of variables

#### 3.2.1 Dependent variable

The numbers of likes (NL), comments (NC), and forwards (NF) of the megacity government microblogs measure public participation behavior (see Table 3). The ‘like’ behavior in microblogs shows users’ approval or preference for the information published, and it is the public participation behavior with the lowest participation level. Compared with the ‘like’ behavior, ‘comment’ is a deeper expression of user participation. Generally, comments capture two things: one is the attitude of the reviewer toward the dynamic, that is, approval or opposition. Another is the familiarity of reviewers and publishers, to some extent. ‘Comment’ is a behavior with a moderate participation level (34). Compared with the ‘comment’ behavior, the ‘forwarding’ behavior is a deeper expression. A ‘Forward’ means that users want more people to see their attitude toward a specific microblog; it is the highest level of participation.

**Table 3:** Description of variables.

Type	Variable name	Variable measurement
Dependent variable	Number of likes (NL)	Number of likes by users
	Number of comments (NC)	Number of comments by users
	Number of forwards (NF)	Number of times forwarded by users
Independent variable	Content richness (CR)	The richness of videos, pictures, and words involved in microblogging
	Content interactivity (CI)	Number of #, @, and hyperlinks contained in the microblog content
	Content length (CL)	Number of words in the microblog content
	Number of fans (NF)	Number of followers of the microblog account
	Update frequency (UF)	Ratio of the number of microblogs released by the microblog account to the number of days registered
Regulatory variable	New confirmed cases (NCC)	The number of new confirmed cases of COVID-19 in the city on T-1

#### 3.2.2 Independent variable

Regarding content richness (CR), the sum of the values of three forms of microblog content: video (value of 3), pictures (value of 2), and words (value of 1) measures the content richness. For content interactivity (CI), the value of a microblog that contains the ‘#’ and ‘@’ symbols and a hyperlink is 3; that with any two is 2; and that with anyone is 1. The value for microblogs without any symbol function is 0. Content length (CL) measures the number of words in the microblog content. The number of fans (NF) measures the number of followers of the government microblog account. The update frequency (UF) is the proportion of the number of microblogs to the number of registered days.

#### 3.2.3 Regulatory variable

This study considered the urban epidemic characteristics and measured the regulatory variable by the number of new confirmed cases (NCC) in each city. The number of new cases released on T-day reflects the number of cases of COVID-19 infection in the city within 24 h on T-1.

### 3.3 Research methods

To analyze the influential factors and paths of public participation behavior of megacity government microblogs, this study applied regression analysis and qualitative comparative analysis (QCA). The software Stata 16.0 was used for the regression analysis. The regression analysis method helps reveal the influence of relevant factors on public participation in terms of statistical significance. However, considering the possible differences in government microblogs in various cities, it is necessary to explore further different combinations of the influential factors. Therefore, the software fsQCA 3.0 was used for QCA to examine the configurations of these influential factors.

## 4 Correlation and regression analysis

### 4.1 Correlation analysis

The correlation analysis of the variables was conducted using Stata16.0 software, and the results are presented in Table 4. The examination revealed that the correlation coefficients among the independent variables were consistently below 0.65, and all VIF values were less than 3, meeting the indicator requirements. Therefore, the study remains unaffected by multicollinearity.

**Table 4:** Correlation analysis.

Variable	1	2	3	4	5	6	7	8	9
1 Number of likes	1								
2 Number of comments	0.849^***^	1							
3 Number of forwards	0.730^***^	0.791^***^	1						
4 Content richness	−0.021^*^	−0.040^***^	−0.073^***^	1					
5 Content interactivity	0.031^**^	0.055^***^	0.028^**^	0.074^***^	1				
6 Content length	0.046^***^	0.041^***^	0.049^***^	0.052^***^	0.016	1			
7 Number of fans	0.028^**^	0.091^***^	0.082^***^	−0.260^***^	0.243^***^	−0.074^***^	1		
8 Update frequency	0.016	0.002	−0.012	0.001	0.023^*^	0.023^*^	−0.186^***^	1	
9 New confirmed cases	0.013	0.070^***^	0.060^***^	−0.254^***^	0.129^***^	0.022^**^	0.196^***^	−0.034^***^	1

*, **, and *** denote significance at the 10, 5, and 1% levels, respectively.

In this study, the dependent variable represents non-negative counting data; therefore, the negative binomial regression model was selected for regression analysis.

### 4.2 Central route regression

Regarding microblog content characteristics, Columns (1)–(3) of Table 5 reveal that the coefficient of content richness is significantly negative, and the secondary coefficient is positive; therefore, there is a significant positive U-shaped relationship between content richness and the number of likes, comments, and forwards of megacities’ government microblogs. The calculations also show that the thresholds of content richness are 3.47, 3.72, and 3.96, meaning that when less (more) than the threshold, an increasing content richness reduces (increases) public participation behavior. Thus, H1 is verified. Columns (4)–(6) of Table 5 show that content interactivity has a positive impact on the numbers of likes, comments, and forwards, meaning that every unit of increase in content interactivity increases the number of likes by 0.3806 times; of comments by 0.6373 times; and forwards by 0.3398 times. In addition, content interactivity has the largest positive impact on the number of comments; hence, H2 is verified. Columns (7)–(9) of Table 5 show that content length has a positive impact on the number of likes, comments, and forwards, and the microblog content length has the largest positive impact on the number of comments; thus, H3 is verified.

**Table 5:** Regression analysis of the central route.

Variable	Likes (1)	Comments (2)	Forwards (3)	Likes (4)	Comments (5)	Forwards (6)	Likes (7)	Comments (8)	Forwards (9)
Content richness	−0.3855^***^ (−6.49)	−0.6615^***^ (−6.66)	−1.3064^***^ (−15.91)						
Content richness^2^	0.0556^***^ (4.72)	0.0889^***^ (4.58)	0.1650^***^ (9.90)						
Content interactivity				0.3806^***^ (19.97)	0.6373^***^ (22.97)	0.3398^***^ (13.16)			
Content length							0.0002^***^ (5.51)	0.0005^***^ (8.04)	0.0002^**^ (2.49)
Constant	1.8222 (13.17)	2.7364 (13.43)	4.0542 (24.63)	0.9801^***^ (11.12)	0.6657^***^ (6.28)	1.6574^***^ (16.63)	1.1511^***^ (11.42)	0.9620^***^ (5.50)	1.5826^***^ (11.62)
Log likelihood	−29073.61	−23760.07	−23412.11	−28888.58	−23479.94	−23652.83	−29093.32	−23768.96	−23736.23
Prob>chi2	0	0	0	0	0	0	0	0	0
N	6,677	6,677	6,677	6,677	6,677	6,677	6,677	6,677	6,677

**, and *** denote significance at the 5, and 1% levels, respectively.

### 4.3 Peripheral route regression

Regarding the characteristics of the microblog publisher, columns (1)–(3) of Table 6 show that the number of followers of the publisher has a significant positive impact on the degree of public participation behavior, which verifies H4. This means that the more fans the government microblog platform attracts, the better the public participation behavior. Columns (4)–(6) show that the frequency of microblog updates significantly negatively affects the degree of public participation behavior. Specifically, every unit of increase in microblog update frequency decreases the number of likes by 0.0683 times; comments by 0.0590 times; and forwards by 0.0379 times. Thus, H6 is verified. The high frequency of government microblogging indicates that the information provided by the microblog is complex and redundant. The public pays attention to the government microblog itself to obtain more information, but if the government microblog releases excessive information, the public may not know what to pay attention to, which reduces the public’s feedback.

**Table 6:** Regression analysis of the peripheral route.

Variable	Likes (1)	Comments (2)	Forwards (3)	Likes (4)	Comments (5)	Forwards (6)
Number of fans	0.6203^***^ (24.65)	0.9124^***^ (24.68)	1.3917^***^ (44.26)			
Update frequency				−0.0683^***^ (−23.45)	−0.0590^***^ (−17.05)	−0.0379^***^ (−12.31)
Constant	−8.3705^***^ (−22.28)	13.0576^***^ (−25.39)	−19.9543^***^ (−44.08)	2.7345^***^ (25.87)	1.5246^***^ (11.56)	1.1417^***^ (8.31)
Log likelihood	−28784.98	−23409.00	−22583.18	−28828.03	−23642.92	−23652.75
Prob>chi2	0	0	0	0	0	0
N	6,677	6,677	6,677	6,677	6,677	6,677

*** denotes significance at the 1% levels.

### 4.4 Regulatory variable regression

Table 7 shows that newly confirmed cases play a regulatory role in the relationship between public participation behavior and its influential factors.

**Table 7:** Analysis results of the regulatory variable.

Variable	Likes	Comments	Forwards
Content richness (Content richness × New confirmed cases)	−0.2846^***^ (0.0006^***^)	−0.3228^***^ (0.0012^***^)	−1.0607^***^ (0.0008^***^)
Content richness^2^ (Content richness^2^ × New confirmed cases)	0.0433^***^ (−0.00008^***^)	0.0473^***^ (−0.0002^***^)	0.1315^***^ (−0.00007^***^)
Content interactivity (Content interactivity × New confirmed cases)	0.3432^***^ (0.0002^***^)	0.5675^***^ (0.0005^***^)	0.2326^***^ (0.0006^***^)
Content length (Content length × New confirmed cases)	0.0002^***^ (8.75e-07^***^)	0.0003^***^ (2.27e-06^***^)	0.00004 (2.56e-06^***^)
Number of fans (Number of fans × New confirmed cases)	0.5699^***^ (0.00003^***^)	0.8109^***^ (0.00006^***^)	1.2815^***^ (0.00005^***^)
Frequency of updates (Updates frequency of × New confirmed cases)	−0.0688^***^ (0.00003^***^)	−0.0628^***^ (0.00004^***^)	−0.0409^***^ (0.00004^***^)
*N*	6,677	6,677	6,677

Figures in brackets are coefficients of the interaction term between the independent and regulatory variable.

Concerning microblog content characteristics, new confirmed cases make the quadratic coefficient of content richness remain significantly positive, that is, the positive U-shaped relationship between content richness and public participation behavior remains significant. The calculation shows that the positive U-shaped curve becomes smoother if the number of confirmed cases increases, and the high number of confirmed cases makes the threshold value of content richness and numbers of likes and forwards shift to the left while the threshold value of the number of comments shifts to the right. Moreover, the interaction items of content interactivity and new cases have a significant positive impact on the degree of public participation, indicating that new confirmed cases significantly and positively regulate the relationship between content interactivity and public participation behavior. Furthermore, the interaction items of content length and new cases have a significant positive impact on the number of likes and comments but have no significant impact on the number of comments. Therefore, H7 is partially verified.

Regarding the characteristics of microblog publishing, the coefficients of interaction items are significantly positive, indicating that newly confirmed cases significantly and positively regulate the relationship between microblog publishing characteristics and public participation. H8 is thus verified.

### 4.5 Grouping analysis

The time feature of microblog publishing and the feature of microblog semantics were further grouped to explore the impact of the central and peripheral routes. The release time was divided into six groups: midnight (0:00–6:00), morning (6:00–12:00), noon (12:00–14:00), afternoon (14:00–18:00), evening (18:00–21:00), and night (21:00–24:00). Semantic features were divided into emotional and rational intensity. Emotional intensity, calculated using Python’s SnowNLP package, shows the emotional value of the microblogging content. The closer this value is to 1, the more positive the microblog content. For rational intensity, —calculated according to the sum of the times the following words were mentioned in Weibo: ‘epidemic’, ‘COVID-19’, ‘pneumonia’, ‘confirmed diagnosis’, ‘case’, ‘dynamic zeroing’, and ‘vaccine’—the higher the frequency, the more scientific, objective, and reasonable the Weibo content is.

In Table 8, the samples are divided into six groups according to the publishing time of the microblog. Regarding the microblog content characteristics, Table 8 shows that the positive U-shaped relationship between the content richness of microblogs published at noon, afternoon, evening, and night and public participation behavior remains significant, but a reverse U-shaped relationship is shown between the content richness of microblogs published in the morning and public participation behavior. Table 8 further shows that the content interactivity of microblogs published in any period is positively related to public participation. In the morning, the content length has a positive impact on public participation, but at noon, the impact is significantly negative. Regarding the characteristics of microblog publishers, the number of fans has no significant impact on the comments only in the early morning, whereas the frequency of microblog updates positively affects public participation in the morning, noon, and night. The update frequency negatively affects public participation behavior in the morning, afternoon, and evening. In general, microblogs in the morning and night, microblog content characteristics, and microblog publisher characteristics lead to different levels of public participation behavior.

**Table 8:** Analysis of microblog publishing and time groupings.

Variable	Likes	Comments	Forwards	Likes	Comments	Forwards
	Midnight			Morning		
Content richness	−0.4388	0.3239	−0.0768	0.7886^***^	1.4864^***^	0.8256^***^
Content richness^2^	−0.0460	−0.2616	−0.1302	−0.0451^***^	−0.0903^***^	−0.1083^***^
Content interactivity	0.8349^***^	1.1927^***^	0.9955^***^	0.7005^***^	1.2817^***^	0.6296^***^
Content length	−0.0009	−0.0009	−0.0011	0.0004^***^	0.0008^***^	0.0002^**^
Number of fans	0.6095^*^	0.7815	1.0184^***^	0.6342^***^	1.0713^***^	1.4153^***^
Update frequency	−0.0734	−0.0608	−0.0629	−0.0640^***^	−0.0350^***^	−0.0255^***^
	Noon			Afternoon		
Content richness	−0.7598^***^	−0.6383^***^	−1.6550^***^	−1.1052^***^	−1.8374^***^	−2.2982^***^
Content richness^2^	0.1264^**^	0.0457^***^	0.2336^***^	0.1755^***^	0.2968^***^	0.3422^***^
Content interactivity	0.4096^***^	0.6207^***^	0.4970^***^	0.2544^***^	0.3536^***^	0.0968^*^
Content length	−0.00001^***^	−0.0001^***^	−0.0003	0.0001	−0.00004	−0.00004
Number of fans	0.8912^***^	0.9887^***^	1.7344^***^	0.6503^***^	0.9109^***^	1.4380^***^
Update frequency	−0.0794^***^	−0.0769^***^	−0.0752^***^	−0.0704^***^	−0.0741^***^	−0.0461^***^
	Evening			Night		
Content richness	−0.6257^***^	−0.9486^***^	−1.6242^***^	−1.4747^***^	−2.1855^***^	−2.5006^***^
Content richness^2^	0.0842^***^	0.1238^***^	0.2146^***^	0.2392^***^	0.3433^***^	0.3672^***^
Content interactivity	0.1436^***^	0.1702^***^	0.0169	0.5552^***^	0.6920^***^	0.7237^***^
Content length	−0.00006	−0.00003	0.0001	−0.0003^**^	−0.0007^***^	0.0005^***^
Number of fans	0.3453^***^	0.4321^***^	0.8948^***^	1.0276^***^	1.3711^***^	1.8653^***^
Update frequency	−0.0655^***^	−0.0727^***^	−0.0346^***^	−0.1100^***^	−0.0925^***^	−0.1551^***^

*, **, and *** denote significance at the 10, 5, and 1% levels, respectively.

**Figure 3:**
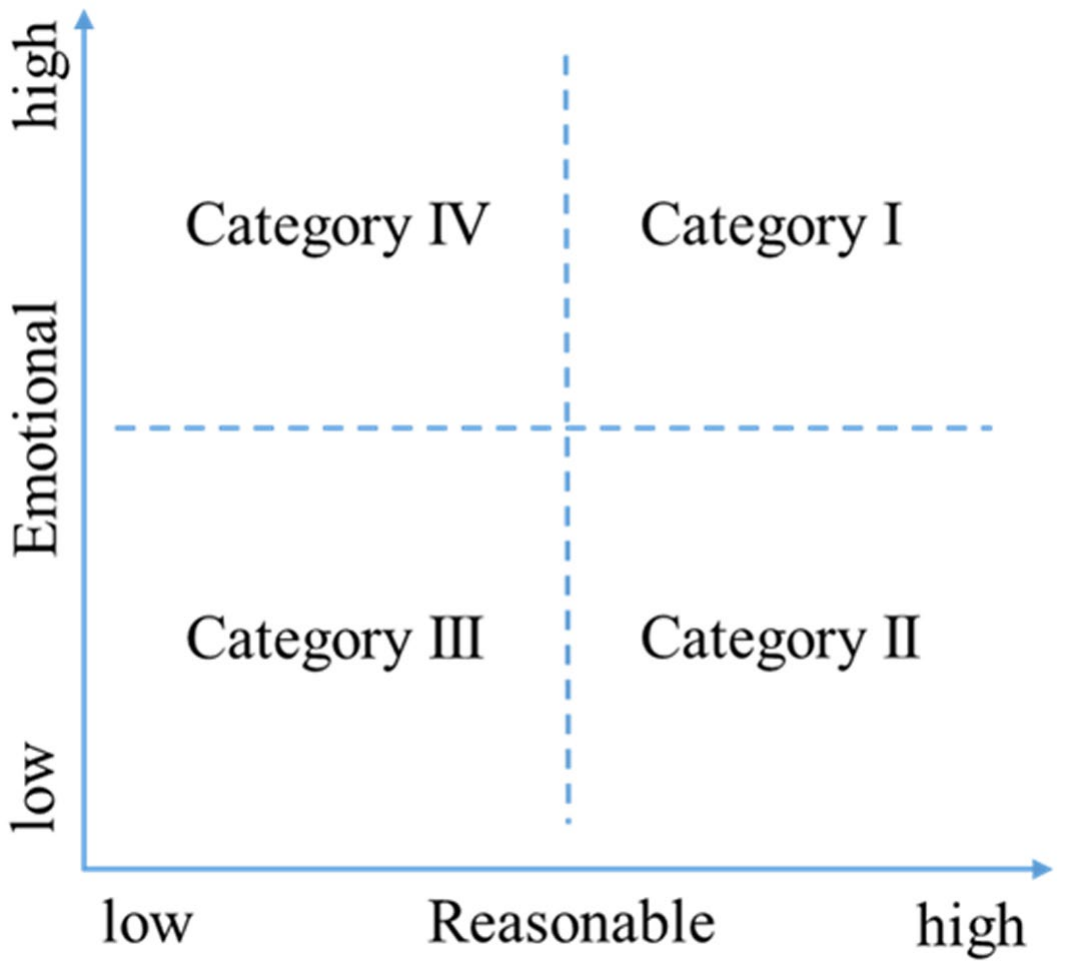
Categories of semantic features.

According to the semantic features of the microblogs, the samples were divided into four categories from two dimensions of the microblog: reasonable intensity and emotional intensity. As Figure 3 shows, Category I is labeled emotional and reasonable, for microblogs that not only release rational information but also reflect appropriate emotion. Category II, called ruthless and reasonable, refers to microblog content that is highly rational but with little emotion. Category III, named ruthless and unreasonable, refers to microblog content that is neither sufficiently rational nor sensible. Category IV, labeled as emotional and unreasonable, refers to microblog content that is highly emotional but lacks scientific rationality.

Regarding microblog content characteristics, Table 9 shows that the content richness of microblogs belonging to Categories I–IV has a U-shaped relationship with public participation behavior. The content interactivity of microblogs in all categories has a positive impact on public participation behavior. The content length of microblogs in Categories I and II has a positive impact on public participation, while that of microblogs in Category IV negatively impacts public participation. Regarding the characteristics of microblog publishers, except for the number of fans for Category II, the number of fans for other categories has a significant positive impact on public participation behavior. Moreover, except for Category II, the update frequency of microblogs belonging to other categories has a significant negative impact on public participation behavior.

**Table 9:** Analysis of the microblogs’ semantic grouping.

Variable	Likes	Comments	Forwards	Likes	Comments	Forwards
	Category I			Category II		
Content richness	−0.9561^***^	−1.8264^***^	−1.5075^***^	0.2939	0.5108^**^	−0.3543
Content richness^2^	0.1168^***^	0.2306^***^	0.1554^***^	−0.0244	−0.0464	0.0329
Content interactivity	0.4569^***^	0.7545^***^	0.36455^***^	0.5020^***^	0.9669^***^	0.0846^**^
Content length	0.0010^***^	0.0012^***^	0.0010^***^	0.0006^***^	0.0012^***^	0.0003^***^
Number of fans	0.8650^***^	1.2832^***^	1.5215^***^	−0.1501^**^	−0.0078	0.3866^***^
Update frequency	−0.0688^***^	−0.0679^***^	−0.0291^***^	−0.0469^***^	−0.0152	−0.0083
	Category III			Category IV		
Content richness	−1.1356^***^	−2.1555^***^	−2.4201^***^	−1.8812^***^	−1.3116^***^	−2.0736^***^
Content richness^2^	0.2001^***^	0.4033^***^	0.3966^***^	0.1131^***^	0.1572^***^	0.2791^***^
Content interactivity	0.3259^***^	0.5207^***^	0.3565^***^	0.3113^***^	0.5219^***^	0.4951^***^
Content length	0.00009	−0.0001	0.00009	−0.0005^**^	−0.0012^***^	−0.0010^**^
Number of fans	0.8628^***^	1.2327^***^	1.7145^***^	0.9234^***^	1.3751^***^	1.9817^***^
Update frequency	−0.0760^***^	−0.0753^***^	−0.1055^***^	−0.0863^***^	−0.0947^***^	−0.1018^***^

**, and *** denote significance at the 5, and 1% levels, respectively.

## 5 Qualitative comparative analysis

As a destination can be reached via various pathways, and outcomes can occur in various ways, explained by different combinations of independent variables (35), this study explored these influential factors’ different configurations using the QCA method.

The overall solution coverage of the seven configuration paths with a high number of likes is 73%, and the overall solution consistency is 0.77. Table 10 shows that Path 1 is characterized by content interactivity * number of fans *update frequency, with coverage of 0.5. This result indicates that to get more likes, the government account needs to have the core conditions of high fans and high update frequency, and the peripheral condition of content interactivity. Path 7 is characterized by content richness * content interactivity * content length * and number of fans, with a coverage rate of 0.3, which is second only to Path 1. This result indicates that if the core conditions are content length and number of fans, and the peripheral conditions are content richness and content interactivity, the government account will be highly liked.

**Table 10:** Configurations for achieving a high number of likes.

Configuration	P1	P2	P3	P4	P5	P6	P7
Content richness		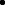	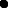	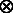	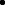	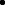	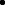
Content interactivity	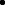	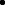	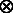	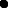	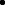		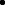
Content length				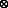	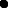	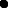	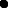
Number of fans	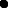	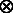	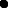	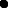		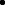	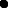
Update frequency	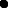	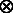	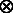		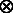	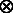	
Raw coverage	0.50	0.30	0.32	0.42	0.35	0.31	0.30
Unique coverage	0.05	0.03	0.01	0.01	0.00	0.00	0.00
Consistency	0.81	0.87	0.80	0.80	0.83	0.80	0.79

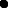
, denotes core casual condition (present); 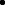, peripheral casual condition (present); 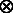, core casual condition (absent); and 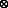, peripheral casual condition (absent). Blank spaces indicate ‘do not care’.

The overall solution coverage of the three configuration paths with a high number of comments is 0.67, and the overall solution consistency is 0.79. Table 11 shows that Path 1 is content interactivity * number of fans * update frequency, with the highest coverage of 0.61. This result indicates that when the number of fans is high, the update frequency is high, and when the content is interactive, the number of comments can be high. Path 2 shows that without a high number of fans and update frequency, government microblogs with high content richness and high content interactivity have a high number of comments. Path 3 shows that without content richness and content length, government microblogs with high content interactivity and a high number of fans have a high number of comments.

**Table 11:** Configurations for achieving a high number of comments.

Configuration	P1	P2	P3
Content richness		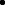	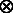
Content interactivity	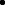	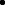	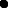
Content length			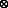
Number of fans	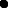	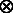	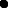
Update frequency	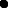	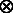	
Raw coverage	0.52	0.30	0.43
Unique coverage	0.10	0.12	0.02
Consistency	0.82	0.85	0.80

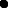
, denotes core casual condition (present); 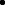, peripheral casual condition (present); 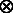, core casual condition (absent); and 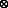, peripheral casual condition (absent). Blank spaces indicate ‘do not care’.

The overall solution coverage of the four configuration paths with a high number of forwards is 0.8, and the overall solution consistency is 0.71. Table 12 shows that Path 2 has high content interactivity * high number of fans and the highest coverage of 0.61. This result indicates that when the number of fans is high and the content is interactive, the microblogs released by the government microblog account have a high number of forwards. Path 1 has high content richness and * a high number of fans, with a coverage rate of 0.51, second only to Path 2. This result indicates that when the government account has a large number of fans and the microblog is highly interactive, the public actively forwards the microblogs. The content length and microblog update frequency of Paths 3 and 4 are the core missing conditions, indicating that even if the content of the microblog is not long and the updating is not frequent, as long as Path 3 has high content richness and Path 4 has a high number of fans, high forwards can be obtained. These results show that the number of fans is not a necessary condition; it is the core existence condition in the three paths (P1, P2, and P4), indicating that the number of fans is an important factor affecting the high number of forwards.

**Table 12:** Configurations for achieving a high number of forwards.

Configuration	P1	P2	P3	P4
Content richness	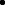		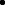	
Content interactivity		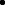		
Content length			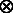	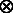
Number of fans	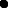	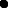		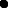
Update frequency			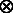	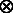
Raw coverage	0.51	0.61	0.41	0.50
Unique coverage	0.02	0.09	0.06	0.05
Consistency	0.79	0.74	0.77	0.81

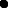
, denotes core casual condition (present); 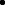, peripheral casual condition (present); 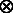, core casual condition (absent); and 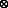, peripheral casual condition (absent). Blank spaces indicate ‘do not care’.

## 6 Conclusions and recommendations

### 6.1 Conclusion

Three primary conclusions are summarized below.

First, concerning the central route, a significant positive U-shaped relationship exists between the content richness of a government microblog and public engagement metrics such as likes, comments, and forwards. Different from a study that indicated a negative impact (8), this study on epidemic-related microblogs reveals a U-shaped relationship between microblog content richness and public participation behavior. Notably, the content richness of pictures occupies the bottom of the ‘U’ shape, positioning it between plain text and video. This U-shaped relationship aligns with the conclusion in the existing literature (18) suggesting that plain text can affect public participation more than pictures. Moreover, this relationship is significant during times of the day noon, afternoon, evening, and night. Content interactivity has a significant positive impact on likes, comments, and forwards, and this positive relationship exists at any time of the day. Content length has a significant positive impact on public likes, comments, and forwards, and this positive relationship is most significant in the morning.

Second, from the peripheral route, the number of fans and update frequency of government microblogs in megacities have a significant impact on public likes, comments, and forwards. In the grouping analysis, the number of fans has a positive impact on public participation behavior in all periods except at midnight, whereas the frequency of microblog updates negatively impacts public participation behavior from morning to night. In addition, for highly emotional microblogs, content characteristics and publisher characteristics have a significant impact on public participation behavior.

Third, different from the research on the data collected by the questionnaire (1, 16), this paper integrates the epidemic data and microblog data, revealing the interaction mechanism between microblog and epidemic. In terms of the regulatory effect, new confirmed cases positively regulate the impact of microblog content and publisher characteristics on public participation behavior.

Fourth, the factors influencing public participation in the government microblogs of different cities may vary. This study further examined the different configurations of these influential factors by applying the QCA method. A configuration of high fans, high update frequency, and high content interactivity can get a high number of likes and comments. By contrast, a configuration of high content interactivity and a high number of fans can get a high number of forwards.

### 6.2 Recommendations

Recommendations are proposed regarding the content format of government microblogs, government microbloggers, and the release of epidemic-related microblogs.

First, the content and publishing form of government microblogs in megacities should be considered carefully. These microblogs should disseminate information through text or video formats to assist the public in problem-solving and knowledge enhancement. Managing content length appropriately is crucial, considering that richer information may attract more users. Longer texts can convey more information, thereby promoting public participation. Megacities should optimize the interactive features of government microblogging, leveraging social media functions, particularly the symbols ‘@’ and ‘#’. The government’s responsiveness to citizens’ concerns can enhance interaction and, thus improve public participation.

Second, megacities should enhance the influence of government microblogging on social platforms. The accumulated number of followers forms the foundation for public engagement with government microblogs. Diversified forms should be employed to attract more followers. The greater the activity and richness of the government microblog’s content, the higher the public’s acceptance. To further improve public participation, megacities should increase the frequency of microblogging while ensuring that the content is of high quality and meaningful.

Third, government microblogs in megacities should promptly disseminate information on epidemic prevention and control. This study demonstrates that during severe outbreaks, microblogs promote public participation. In such situations, government microblogs should prioritize releasing information about epidemic prevention and advising the public on self-protection measures. Government microblogs should also pay attention to the timing of microblog releases, aiming to minimize content length during noon and night. Public engagement is more likely with concise information and meaningful information, avoiding lengthy and complex details. Furthermore, released information should consider emotional impact and maintain rationality, not only describing current issues but also evoking public emotions.

## 7 Limitations

In this study, economic and social development data of megacities were excluded from the model. Future research should optimize the model further, incorporating additional research variables based on central and peripheral routes. Additionally, expanding the sample size by including data from small and medium-sized cities or cities from other countries would enable comparative analyses.

## Data availability statement

The raw data supporting the conclusions of this article will be made available by the authors, without undue reservation.

## Author contributions

PS: Conceptualization, Data curation, Funding acquisition, Supervision, Writing – review & editing. ML: Methodology, Writing – original draft.
